# Domain-general cognitive functions fully explained growth in nonsymbolic magnitude representation but not in symbolic representation in elementary school children

**DOI:** 10.1371/journal.pone.0228960

**Published:** 2020-02-11

**Authors:** Yulia Kuzmina, Tatiana Tikhomirova, Irina Lysenkova, Sergey Malykh

**Affiliations:** 1 Department of Psychology, Lomonosov Moscow State University, Moscow, Russia; 2 Psychological Institute of Russian Academy of Education, Moscow, Russia; 3 Department of Psychology, Kyrgyz-Russian Slavic University, Bishkek, Kyrgyzstan; French National Center for Scientific Research (CNRS) & University of Lyon, FRANCE

## Abstract

In this study, we aimed to compare developmental changes in nonsymbolic and symbolic magnitude representations across the elementary school years. For this aim, we used a four-wave longitudinal study with a one-year interval in schoolchildren in grades 1–4 in Russia and Kyrgyzstan (N = 490, mean age was 7.65 years at grade 1). The results of mixed-effects growth models revealed that growth in the precision of symbolic representation was larger than in the nonsymbolic representation. Moreover, growth in nonsymbolic representation was fully explained by growth in fluid intelligence (FI), visuospatial working memory (VSWM) and processing speed (PS). The analysis demonstrated that growth in nonsymbolic magnitude representation was significant only for pupils with a high level of FI and PS, whereas growth in precision of symbolic representation did not significantly vary across pupils with different levels of FI or VSWM.

## Introduction

Humans are equipped with the ability to estimate numerosity in both symbolic and nonsymbolic formats. The ability to estimate numerosity in nonsymbolic format implies the ability to compare, order, add or subtract quantities of object without using symbols (digits) or verbal forms (number words). The ability to process and manipulate numerosity without using symbols and counting is commonly referred to as the Approximate Number System (ANS) [1].

The ANS is believed to be evolutionarily ancient and shared by non-human animals, primates and humans [2, 3]. In particular, it has been demonstrated that fish, birds, and primates can discriminate arrays of objects differing in numerosity [4–6]. Studies of human infants have also shown that 6-month-old babies are able to discriminate arrays of objects in cases in which the ratio between them is 1:2 [7, 8]. It was also found that the ANS was not limited to visual stimulus and that infants were able to discriminate arrays of sounds [7, 9]. Moreover, discrimination of visual and audio stimulus revealed similar patterns of success and failures.

Although the ANS is an evolutionarily ancient ability, it has been demonstrated that the ANS is modestly heritable, with individual differences being largely explained by non-shared environmental influences (e.g., [10]. The ANS was discussed as a “fitness” trait–a trait that has limited heritability and for which genes do not play a large role in determining individual differences.

As a result of observations on tests of nonsymbolic discrimination in animals, infants, children and adults, two core features of the ANS have been hypothesized, namely, the ratio effect and the size effect, which reflect the imprecision of the ANS. The ratio effect means that the precision of discrimination between two arrays depends on the ratio between them [11]. Imprecision in nonsymbolic estimation may increase when the ratio between two arrays of objects that must be discriminated has increased or when arrays have become more similar in numerosity [12, 13]. This ratio dependence can also be described as a Weber fraction–the smallest difference between two arrays that can be reliably identified [14]. The size effect reflects the growing imprecision of nonsymbolic discrimination when the numerosity increases while the ratio between two arrays remains the same [15]. The existence of the ratio and size effects in nonsymbolic discrimination tasks may be explained by the overlapping of Gaussian curves reflecting the internal representation of each numerosity on a mental number line [14, 16].

The accuracy of the ANS in schoolchildren and adults is often measured by various “dots” tests, in which individuals have to compare two sets of dots and decide which set contains more dots [17, 18]. The precision of the ANS in these tests can be measured by the proportion of correct answers or the Weber fraction [e.g., 19]. It has been demonstrated that the proportion of correct answers and the Weber fraction are highly correlated [19, 20] and that among four possible indicators of accuracy of the ANS (proportion of correct answers, *w*, reaction time and numerical ratio effect), the number (proportion) of correct answers had a higher test-retest reliability [19].

Studies have demonstrated that the precision of nonsymbolic representation increases with development [e.g. 21]. Growing precision of the ANS manifested as a decrease in Weber fraction (*w)* [21–23] or an increasing proportion of correct answers in nonsymbolic comparison tasks [24, 25]. The peak of precision in ANS and smallest *w* occur near 30 years of age [21].

There is evidence that education plays a significant role in the development of nonsymbolic representation. Some authors have suggested that the experience of manipulations with number symbols and the acquisition of number knowledge occurring during formal education enhanced the accuracy of nonsymbolic magnitude estimation [26, 27]. In particular, ANS acuity was higher in individuals who had a formal education experience than in individuals without formal education [28, 29]. It was also shown that the precision of nonsymbolic representation may increase after training and feedback [30, 31]. However, some studies demonstrated that experience with formal math was not always associated with growth in ANS precision [24, 27, 32].

There is evidence that ANS serves as foundation of acquisition of symbolic numerosity representation [33, 34]. Unlike ANS, symbolic magnitude representation is more precise and exists only in humans [35, 36]. Symbolic estimation is based on the acquisition of number-system knowledge and counting words [1, 37]. Using number symbols enables the precise estimation of the difference between two numerosities that can be presented in symbolic (digits) or nonsymbolic (sets of dots) formats.

The precision of symbolic magnitude representation might be investigated by variety of tests, including the number line (NL) test. In one version of this test, individuals have to identify the position of a number on a symbolic number line with marked starting and end points [38, 39]. The precision in the NL test is significantly associated with results of different math tests and other tests of symbolic representations [40, 41]. Moreover, NL test results were more closely linked to math achievements than the symbolic magnitude comparison task [40].

Some researchers have suggested that the NL test can measure other abilities in addition to symbolic magnitude representation. In particular, it was suggested that the NL test measured proportional judgement skills or number-numerosity mapping skills or may be confounded with visuospatial skills [39, 42, 43]. In any case, number line estimation is based on symbolic representations and required number system knowledge [39, 44].

For the NL test there exist several indicators of accuracy reflecting the precision of symbolic representation. The first indicator is the deviation of the identified position of a number on a number line from the actual position of the number. The deviation can be divided on scale of estimates to obtain the Percentage Absolute Error (PAE) [45, 46] or used in the absolute term [47]. The second indicator is the pattern of relationships between marked and actual numbers. Several models of relations between actual and marked numbers (e.g., logarithmic, exponential, linear) are calculated, and the model with the largest proportion of explained variance is then selected [45, 48]. For example, at a young age, relations between actual and marked numbers are better described by nonlinear models, such as logarithmic models [49, 50]. It was demonstrated that PAE was a better predictor of math achievement than the model of relations between actual and estimated numbers [40].

The growing precision of symbolic representation in the NL test manifested in an increased precision in the identification position of a number on a number line and a reduction of PAE [51, 52, 41]. In addition, throughout development, the less accurate logarithmic model is replaced by more accurate linear representations of numbers on the number line [45, 48, 53, 54]. Several studies demonstrated that logarithmic and linear models might co-exist in one age depending on the scale of NL [48, 45]. Particularly, the same child might be able to map a linear representation onto the number line on a 0–100 scale and logarithmically on a 0–1000 scale [48].

Most findings have confirmed that precision in symbolic representation in general and in the NL test specifically increased due to experience in formal education and the acquisition of number system knowledge [32, 55, 56]. In particular, it was demonstrated that linear relations between numbers and their placement on a number line were identified only for individuals with experience of formal education [56]. There is also evidence that education has a larger effect on the development of symbolic representation than on the development of the ANS [32, 57]. In particular, at the beginning of formal school education, symbolic number skills developed at a higher rate than nonsymbolic skills [58].

Although the growing precision of nonsymbolic and symbolic representation throughout development was confirmed in variety of studies, there exist some issues regarding the mechanisms of developmental changes in these abilities. The first issue refers to contradictory findings about developmental relations between nonsymbolic and symbolic representations. Many authors have suggested that acquisition of symbolic magnitude representations occurred through mapping number symbols onto ANS and that growing precision in nonsymbolic representation enhances the acquisition of symbolic skills [33, 59].

At the same time, several findings have contradicted this hypothesis. Some studies demonstrated that nonsymbolic and symbolic magnitude representations are distinct systems that have no significant correlation at least in early school-aged children [38, 58, 60, 61]. Others have postulated that a symbolic representation system refines nonsymbolic representation rather than the opposite [62, 63]. Hypotheses of bidirectional relationships between nonsymbolic and symbolic representations have also been suggested [64, 65]. Hence, there exist contradictions regarding developmental relations between symbolic and nonsymbolic representations.

The second issue refers to the lack of knowledge regarding the extent to which developmental changes in precision of symbolic and nonsymbolic representation are distinct from developmental changes in general cognitive functions such as fluid intelligence, processing speed or working memory. It was shown that fluid intelligence and working memory are associated with the precision of symbolic and nonsymbolic magnitude representation [66, 67]. Particularly, nonsymbolic representation was predicted by the central executive component of WM (the modality-free component, which controls and coordinates the functioning of two “slave” systems: phonological loop and visuospatial sketchpad). The symbolic representation was predicted by the central executive and visuospatial sketchpad (the component of WM that is involved in processing and maintaining visuospatial images) [66]. There is also evidence that fluid intelligence is correlated with precision of symbolic magnitude representation [68, 69].

There is considerable evidence that general cognitive abilities such as fluid intelligence, processing speed and VSWM improved throughout development [70, 71]. Behavioral genetic studies suggested the existence of generalist genetic overlaps between the ANS, math achievement and domain-general cognitive factors [72]. Improvement in general cognitive abilities may lead to growing precision of nonsymbolic and symbolic representations. Increasing precision in numerosity representations may be explained by improvement in general cognitive abilities.

In addition, it is unknown how progress in symbolic and nonsymbolic representations varies for children depending on their level of general cognitive abilities. The rate of changes in precision of nonsymbolic and symbolic representation might vary for children depending on their level of fluid intelligence, working memory or processing speed.

The current study had three aims. The first aim was to estimate growth trajectories and developmental relations between nonsymbolic and symbolic magnitude representations across four years of elementary school. We used data from a four-year longitudinal study of elementary schoolchildren who participated in an ongoing longitudinal project CLASS (Cross-cultural Longitudinal Analysis of Student Success). To measure precision of nonsymbolic representation, a nonsymbolic comparison task was used, whereas the NL test was used to measure the precision of symbolic magnitude representation.

The second aim was to estimate the extent to which four-year changes in domain-general cognitive functions, such as fluid intelligence, processing speed and visuospatial working memory, are related to developmental changes in the precision of nonsymbolic and symbolic magnitude representations. The third aim was to evaluate whether growth in the precision of nonsymbolic and symbolic magnitude representations varied across pupils depending on their level of general cognitive abilities.

## Materials and methods

### Participants

Data for this study were collected from 612 schoolchildren in grades 1–4 from 17 classes in two schools from Russia and Kyrgyzstan. The schools were equal in terms of rating within their region (e.g., the ratio of the average school scores on the final state mathematics examination to the average scores for the region), teacher characteristics (e.g., the ratio of teachers with higher pedagogical education to the total number of teachers, teachers’ experience and age) and curriculum (the mathematics and Russian language programs at the primary, secondary and high schools were the same).

In both countries, all children studying in the first grade at the start of the longitudinal project participated in the study. The reasons for nonparticipation were illness or absence from school on the date of testing. The study received approval from the Ethics Committee of the Psychological Institute of the Russian Academy of Education. Parental and participant informed consents were obtained prior to data collection.

As at least three time points are necessary to carefully estimate developmental trajectories and development relationships [73, 74], only data from pupils who participated three or four times were analyzed. The patterns of missing data in the sample were tested, and the MCAR (missing completely at random) assumption was confirmed by Little’s MCAR test [75]. This test was nonsignificant (chi-square distance = 272.51, df = 248, *p* = 0.14), indicating that the MCAR assumption held. Since the MCAR assumption held and the sample size was sufficient, list-wise deletion can be applied to obtain adequate parameter estimates [76].

The final sample consisted of 490 participants (51% girls), 27% of whom participated three times, and 73% of whom participated four times. The mean age was 7.65 years in grade 1 (SD = 0.37, range 6.42–8.83). The Kyrgyz sample included 262 participants (55% girls). A total of 66% of the participants were Kyrgyz, and 10% were Russian; the remaining participants belonged to other ethnic groups (e.g., Dungan, Uyghur, Kazakh). The Russian sample included 228 participants (46% girls), all of whom were Russian. The Russian and Kyrgyz samples did not differ at the family educational level. The proportion of mothers who had higher education was 50.38% in the Russian sample and 52.65% in the Kyrgyz sample. In both schools, instruction was in Russian.

### Procedure and materials

All measurement waves occurred at the end of the academic year (April-May). All participants were tested in quiet settings within their school facilities by a trained experimenters. All the experimenters strictly used the same protocol with instructions for testing administration across all measurements.

The experiment every year took place in two sessions. During the first session pupils completed tests for estimating symbolic and nonsymbolic magnitude representations, a test for estimation visuospatial working memory and a test for estimation of processing speed. These tests were executed in computer form. The experiment was performed in a computer classroom in groups of 14–15 pupils. Each pupil sat in front of an individual monitor screen approximately 60 cm from the screen and performed the experiment independently. Each computer had a 17” LCD display with a resolution of 1,440–900 pixels and a refresh rate of 60 Hz.

The second session took place in the subsequent 1–2 days. Pupils’ fluid intelligence was estimated via the Raven’s Standard Progressive Matrices (SPM) test, which was performed in paper-and-pencil format. The duration of each session was approximately 40 minutes. The sequence of the tests was the same at each time point.

#### Nonsymbolic magnitude representation

The nonsymbolic comparison test was used to estimate the precision of nonsymbolic magnitude representation at each time point. The participants were presented with arrays of yellow and blue dots in intermixed format and varying in size and number. The task required the participants to judge whether the array contained more yellow or blue dots by pressing the corresponding keys on the keyboard. The stimuli were 150 static pictures, with the arrays of yellow and blue dots varying between 5 and 21 dots of each color and the ratios of the arrays of the two colors falling between 1:3 and 6:7. In each trial, the cumulative area of the set containing more dots was larger. The ratio of the cumulative areas of the two sets (the smallest area divided by the largest area) ranged between 0.30 to 0.99. In all trials, the average size of the yellow dots was equal to the average size of the blue dots.

The presentation order was the same for all participants. The stimulus flashed on the screen for 400 ms, and the maximum response time was 8 seconds. If no answer was given during this time, then the answer was recorded as incorrect, and a message appeared on the screen to encourage the participant to press the space bar to see the next trial. The message disappeared after 20 seconds, and the next trial was displayed only after pressing the space bar. The task contained a set of instructions, a practice trial with two items and an option to repeat the practice.

The accuracy in the ANS test was calculated as the sum of correct answers. Higher values corresponded to a higher precision of nonsymbolic magnitude representation.

#### Symbolic magnitude representation

The number line test was used to estimate the precision of symbolic magnitude representation at each time point. This test was programmed and adapted online from a description obtained from [48, 77]. A line was presented on the screen with a number at the top of the screen. An 11.5-pixel-high vertical mark indicated the start and end of the number line. The left end of the line was marked with a “0”, and the right end was marked with the number “1,000”. The total length of the line was 500 pixels, allowing the line to be correctly displayed on the computer screen. The center of the number line was at the center of the screen. The target number was 0.4 cm in height and placed 3 cm above the center of the number line.

The task required the participants to place the number displayed along the line. In total, 22 numbers were estimated, and these numbers were presented to all participants in the same order at various time points as follows: 246, 179, 818, 78, 722, 150, 366, 122, 738, 5, 147, 938, 18, 606, 2, 34, 754, 100, 56, 163, 486, and 725.

Each pupil could move the mouse to mark the position of the estimated number. The movement of the mouse coincided with the movement of a vertical red line (18.5 pixels) on the number line. When an individual decided to give an answer and mark the position, s/he clicked on the left mouse button.

There was only one practice trial in this test to reduce the effects of training, as training has been shown to positively affect estimation accuracy. It was possible to take breaks. On each screen, there was an option to continue with the task or resume it later.

Accuracy on the NL test was calculated as a difference between the maximum possible deviation (1000) and the mean deviation from the correct position of the number. This transformation was performed to better compare accuracy in the ANS and NL tests. Thus, larger numbers corresponded to a more accurate identification of the position and more precise symbolic representation.

#### Fluid intelligence

Raven’s SPM test was used to estimate FI at each time point. The original version of the test comprises 5 sets—A, B, C, D, and E. Within each set, 12 items progressively become more difficult thus, there were 60 tasks in total [78]. There was no discontinuity rule, and all participants completed all tasks. The accuracy was calculated as a sum of correct answers.

#### Visuospatial Working Memory (VSWM)

The Corsi Block-Tapping test was used to measure VSWM. This test has been programmed and adapted for online administration from the pen and paper version described in [79]. We reduced the number of items per trial from the original version based on an internal validity analysis conducted during previous pilot testing. Participants were presented with a block where cubes glow one at the time in established patterns. The task consisted of reproducing the correct pattern by clicking on the boxes with the mouse.

The test started with 4 items (or tappings of the cubes) in each sequence. There were 2 sequences for each level and 9 levels, resulting in a total of 18 trials. If students completed both or just one correct sequence on a level, the first item of the next level was shown. The test was interrupted when both sequences on a particular level were incorrectly reproduced. The test started with visual instructions and one practice trial of 3 items that could have been repeated until the participant was familiar with the task. During presentation of the stimuli, the box glowed for 1 second. An interval of 1 second separated the glowing of the boxes. On each screen, there was the option to continue with the test or take a break and resume it later. Accuracy was calculated as a sum of correct answers.

#### Processing speed

Processing speed was measured via a reaction time test. The test was programmed online following the procedure described in [80]. In this version, the numbers 1, 2, 3, and 4 appeared 10 times each in a randomized order with random intervals between 1 and 3 seconds. The task consisted of pressing the key corresponding to the number appearing on the screen as fast and accurately as possible. We programmed the task using only one series of numbers that was used with all participants. The numbers appeared 10 times each, the interval of 1 second was repeated 14 times and the interval of 2 and 3 seconds between the presentations was repeated 13 times each. The task started with instructions and a practice trial consisting of 6 items. The practice trial could have been repeated. The time out for responses was 8 seconds. If no response was given during this time, the next trial followed. The mean reaction time for correct answers was calculated as an indicator of PS. Lower reaction times corresponded to higher PS.

### Statistical approach

To estimate growth trajectories for nonsymbolic and symbolic representations, we used the mixed-effect growth approach (ME approach). The ME approach accounts for the fact that individuals are measured repeatedly over time and that parameters are estimated within a multilevel regression framework, while variables that change over time are treated as “nested” in individuals. This approach allows researchers to estimate the average trajectory for the entire sample and individual-specific deviations from the average trajectory for each person. According to this framework, the intercept and the slope may vary across individuals, and this heterogeneity is described by the variance in the intercept and the slope.

We tested several models and used the likelihood ratio test (LR test) to choose the best-fitting model with the ANS and NL accuracy values as outcomes:

1. Baseline model (intercept only). This model estimates the average accuracy for ANS or NL across all grades and implies that there are no changes in ANS or NL.
2. Model 1. Linear growth model with a random intercept and fixed slope. In this model, the time variable is added. The coefficient of the time variable indicates how accuracy changes over time. This model implies that there are inter-individual differences in the ANS or NL at the starting point (grade 1) but that the rate of growth does not significantly differ between individuals (i.e., the slope of the time variable does not significantly vary between participants).
3. Model 2. Model with a random slope of the time variable. This model implies that there are significant differences between individuals in the rate of changes in ANS or NL across the four grades. In this model, the variance in the slope of the time variable and the covariance between individual deviation of the slope and the intercept were estimated. We determined which model (random or fixed slope) better fit the data.
4. Model 3. Nonlinear growth model. We add a time-squared variable to determine whether the model with nonlinear changes better fit the data.

To reach the second research aim, after the best fitting model was selected, we included FI, VSWM and PS as predictors (Model 4). If cognitive predictors explained time changes in ANS or NL accuracy, the value of the coefficient for the time variable would become reduced or the coefficient would become nonsignificant.

Next, in Model 5, NL precision was included as a predictor for ANS accuracy and, vice versa, ANS accuracy was added as a predictor for NL accuracy, to control for possible relations between symbolic and nonsymbolic magnitude representations. We also controlled for between-country differences by adding a country variable (0 = Kyrgyzstan, 1 = Russia).

To achieve the third goal of this study, we tested several models with interactions between time variables and cognitive predictors. In Model 6, we included the interaction between FI and time. In Model 7, we included the interaction between VSWM and time. In Model 8, the interaction between PS and time variable was added. The significance of the interaction terms means that the time changes varied across pupils with different levels of FI or VSWM.

All variables were transformed into Z-scores before being included into the analysis to make the coefficients from the models for two dependent variables comparable. Besides, standardization of variables has been recommended for multilevel regression models with random slopes and interactions (e.g., [81, 82]). Transformation into Z-scores was performed around the sample and time mean. This transformation enabled the time changes in outcomes to be estimated. The analysis was performed using Stata 15.0 software [83].

## Results

### Descriptive statistics

Descriptive statistics for each measure in Grades 1–4 are shown in Table 1.

**Table 1:** Means, standard deviations and ranges for ANS, NL, FI, VSWM and PS.

Variables	Grades	Mean	SD	Min	Max
**ANS (sum of correct answers)**	Grade 1	92.20	14.13	61	124
	Grade 2	95.47	12.99	56	124
	Grade 3	98.79	13.40	63	130
	Grade 4	100.29	13.73	63	131
**ANS (proportion of correct answers)**	Grade 1	.61	.09	.41	.83
	Grade 2	.64	.09	.37	.83
	Grade 3	.66	.09	.42	.87
	Grade 4	.67	.09	.42	.87
**NL**	Grade 1	836.54	96.50	456	968.2
	Grade 2	873.80	83.45	551.3	976.9
	Grade 3	912.78	62.07	501.2	981.7
	Grade 4	930.52	46.13	673.05	980
**FI**	Grade 1	28.16	10.65	4	53
	Grade 2	33.88	9.66	3	54
	Grade 3	38.56	8.13	11	60
	Grade 4	41.66	7.49	12	57
**VSWM**	Grade 1	2.05	1.60	0	7
	Grade 2	2.52	1.77	0	7
	Grade 3	3.38	1.75	0	8
	Grade 4	4.11	1.75	0	9
**PS**	Grade 1	1.14	.30	.33	2.40
	Grade 2	1.01	.28	.52	2.47
	Grade 3	.93	.26	.48	1.92
	Grade 4	.85	.24	.43	2.31

The descriptive statistics results revealed that the sample mean accuracy in the ANS and NL tests increased across the four grades, and the mean value of FI and VSWM scores also increased. Similarly, PS improved, and the mean reaction time decreased from grade 1 to grade 4.

### Developmental trajectories for ANS and NL

The results of mixed-effects growth modeling for the ANS are presented in Table 2.

**Table 2:** Results of mixed-growth modeling for ANS development.

	Baseline model (without predictors)	Model 1 (linear growth, fixed slope)	Model 2 (linear growth, random slope)	Model 3 (non-linear growth)
***Fixed effects***				
**Constant**	.005 (.03)	-.31*** (.04)	-.31*** (.04)	-.36*** (.05)
**Time**		.20*** (.02)	.20*** (.02)	.32*** (.06)
**Time**^**2**^				-.04* (.02)
***Random effects***				
**Intercept variance**	.37	.38	.46	.47
**Residuals**	.63	.56	.49	.48
**Slope variance (time)**			.05	.05
**Covariance between intercept and slope (time)**			-.05	-.05
**Log likelihood**	-2362.37	-2291.65	-2281.98	-2279.42
**LR test**^a^ **(Δdf)**^b^		141.43*** (1)	19.34*** (2)	5.11* (1)

***p < .001

* *p* < .05

^a^ Likelihood ratio test

^b^ Difference in the degrees of freedom

The results of the baseline model demonstrated that the intraclass correlation coefficient (ICC) (proportion of within-individual variance to common variance) was 0.37. The results revealed that model with nonlinear growth and a random slope for the time variable fit the data better than the model with linear changes and a fixed slope. Therefore, there were significant inter-individual differences in the rate of growth for ANS accuracy. The covariance between the individual rate of changes and the intercept that refers to ANS at grade 1 was significant and negative. The negative covariance indicated that the rate of growth in ANS accuracy was lower for individuals who had a higher accuracy at the initial time point (at Grade 1). The quadratic term of the time variable was significant and negative. This data indicated that the rate of growth slowed across grades 1–4.

Next, we estimated growth trajectories for NL accuracy growth (Table 3).

**Table 3:** Results of mixed-growth modeling for NL development.

	Baseline model (without predictors)	Model 1 (linear growth, fixed slope)	Model 2 (linear growth, random slope)	Model 3 (non-linear growth)
***Fixed effects***				
**Constant**	.004 (.03)	-.60*** (.04)	-.60*** (.05)	-.67*** (.05)
**Time**		.39*** (.02)	.39*** (.02)	.58*** (.05)
**Time**^**2**^				-.06*** (.02)
***Random effect***
**Intercept variance**	.24	.29	.84	.84
**Residuals**	.76	.52	.44	.43
**Slope variance (time)**			.05	.05
**Covariance between intercept and slope (time)**			-.21	-.21
**Log likelihood**	-2532.72	-2274.16	-2189.93	-2182.16
**LR test (Δdf)**		517.12*** (1)	168.45*** (2)	15.56*** (1)

***p < .001

The results of the baseline model indicated that the ICC for NL accuracy was 0.24. The results for the NL also revealed that the model with nonlinear growth and a random slope for the time variable fit the data better than the models with linear changes and fixed slopes. Therefore, there were significant inter-individual differences in the rate of growth of NL accuracy. The covariance between individual rate of changes and the intercept that refers to NL at grade 1 was significant and negative. Hence, the rate of growth in NL was higher for individuals who had a higher precision at Grade 1. It should be noted that this covariance was stronger for NL than for ANS. It is possible that initial status in NL and the rate of growth were associated to a greater extent for NL than for ANS. The quadratic term of the time variable was significant and negative. These data indicated that the rate of growth slowed across grades 1–4 for NL accuracy as well.

**Fig 1:**
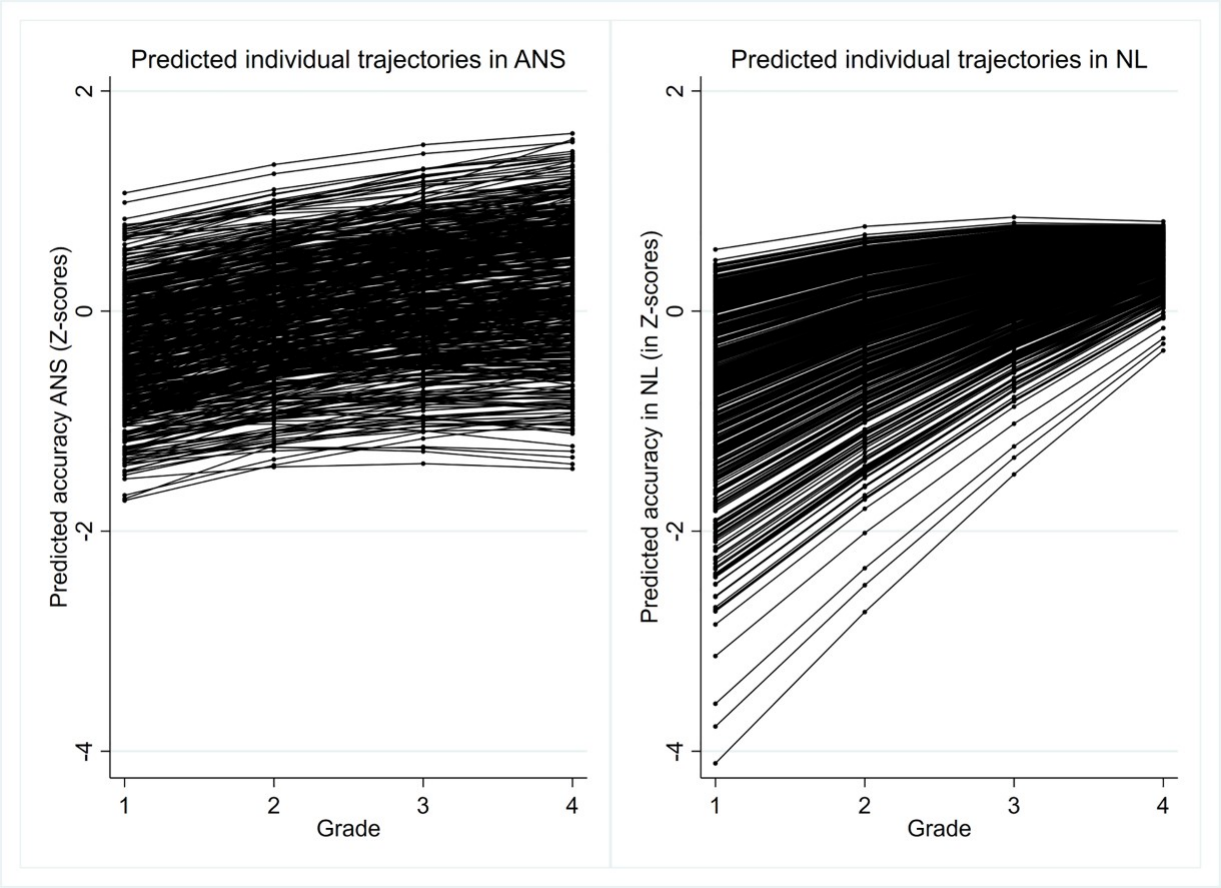
Individual predicted trajectories in ANS and NL growth.

As both ANS and NL variables were transformed into Z-scores, it was possible to consider changes in both variables on one scale measured by standard deviations. Individual predicted trajectories for ANS and NL growth are presented in Fig 1.

Differences existed in patterns of individual changes in ANS and NL. In particular, the development of an NL compensatory pattern was revealed. This pattern was characterized by a diminishing gap between individuals across time and shows that individuals with higher accuracy at Grade 1 demonstrated lower growth. Meanwhile, intra-individual differences in ANS did not significantly change over time.

**Fig 2:**
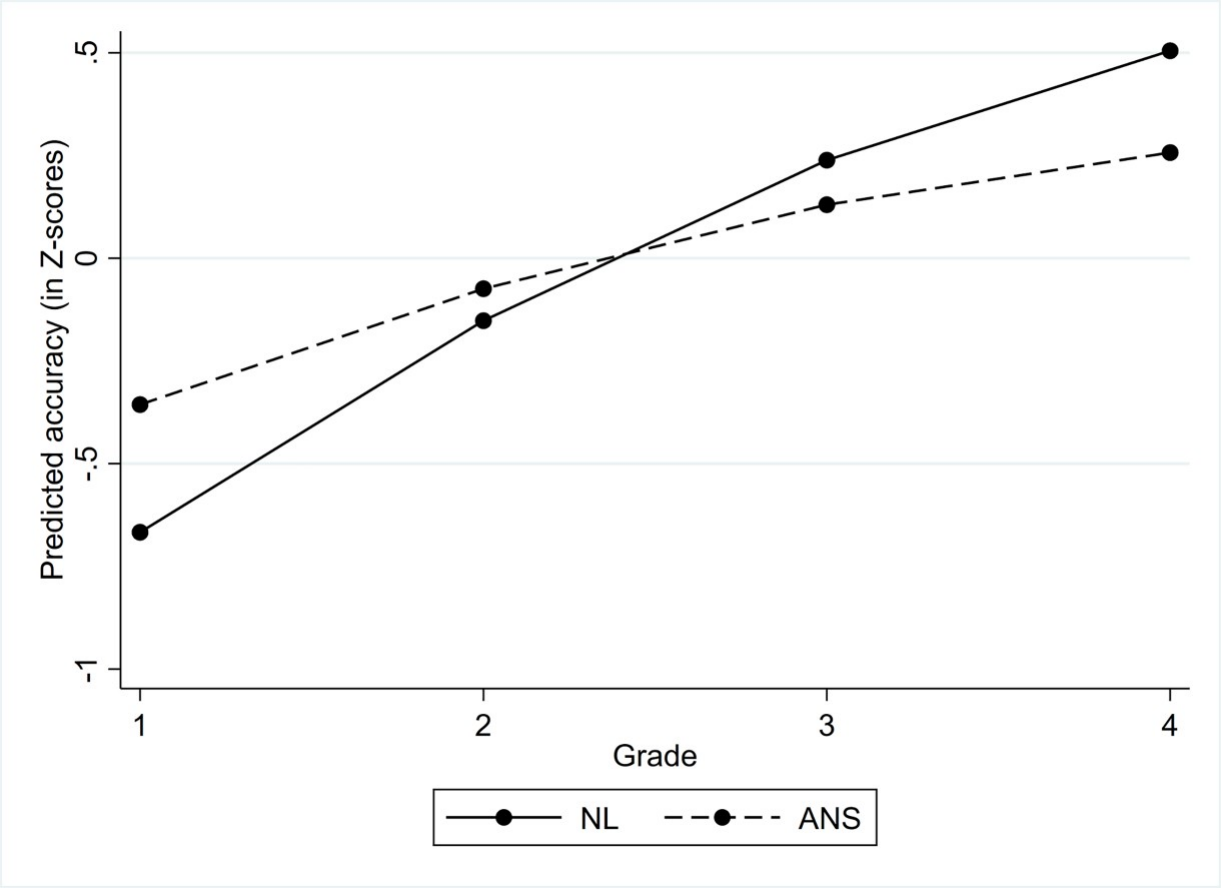
Average developmental trajectories for ANS and NL performance.

Comparison of the average sample developmental changes in ANS and NL performance revealed that NL accuracy increased at a higher rate than the ANS accuracy (Fig 2).

### How cognitive factors are related to growth in ANS and NL performance

Next, we included cognitive predictors in the model with ANS accuracy as the outcome (Table 4, Model 4). We also added NL accuracy and country variable in Model 5.

**Table 4:** Results of mixed-effects analysis for ANS growth and cognitive predictors.

	Model 4 (FI, VSWM, PS added as predictors)	Model 5 (FI, VSWM, PS, NL and country added as predictors)
***Fixed effects***		
**Constant**	-.07 (.05)	-.12 (.07)
**Time**	.11 (.06)	.08 (.06)
**Time**^**2**^	-.02 (.02)	-.02 (.02)
**FI**	.17*** (.03)	.12*** (.03)
**VSWM**	.14*** (.03)	.13*** (.03)
**PS**	-.13*** (.03)	-.10*** (.03)
**NL**		.12*** (.03)
**Country**		.15* (.07)
***Random effects***		
**Intercept variance**	.35	.35
**Residuals**	.49	.49
**Slope variance (time)**	.05	.05
**Covariance between intercept and slope (time)**	-.07	-.07
**Log likelihood**	-2168.67	-2141.51

*** *p* < .001

* *p* < .05

These results revealed that after including cognitive predictors in the model, the coefficients for the time variables became nonsignificant. These results indicated that growth in the ANS became nonsignificant if time changes in FI, VSWM and PS were controlled for. The results of Model 4 also revealed that FI, VSWM and PS were significantly related to ANS accuracy. The results of Model 5 demonstrated that NL accuracy was positively associated with the ANS even when domain-general cognitive factors were controlled for.

The results for estimating the growth in NL accuracy adjusted for the development of FI, VSWM and PS are presented in Table 5.

**Table 5:** Results of mixed-effects analysis for NL growth and cognitive predictors.

	Model 4 (FI, VSWM, PS added as predictors)	Model 5 (FI, VSWM, PS, NL and country added as predictors)
***Fixed effect***		
**Constant**	-.42***(.05)	-.42*** (.06)
**Time**	.38*** (.06)	.38*** (.06)
**Time**^**2**^	-.05** (.02)	-.05** (.02)
**FI**	.18*** (.03)	.17*** (.03)
**VSWM**	.11*** (.02)	.09*** (.02)
**PS**	-.08**(.02)	-.06* (.02)
**ANS**		.11*** (.02)
**Country**		.003 (.05)
***Random effects***		
**Intercept variance**	.69	.71
**Residuals**	.43	.42
**Slope variance (time)**	.05	.06
**Covariance between intercept and slope (time)**	-.19	-.20
**Log likelihood**	-2066.58	-1961.88

***p < .001

**p < .01

* *p* < .05

The results of Model 4 for NL accuracy revealed that cognitive predictors partly explained the growth in NL accuracy. The growth in NL accuracy remained significant after cognitive predictors were added. Among the cognitive predictors, FI had the largest association with NL. Model 5 revealed that ANS was a significant predictor of NL accuracy even when controlling for domain-general predictors.

### How growth in ANS and NL performance varied for pupils with different levels of general ability

To estimate how growth in ANS or NL performance varied for students with different levels of general ability, we included the interaction term between cognitive predictors and the time variable. The results analysis for ANS as outcome are presented in Table 6.

**Table 6:** Results of mixed-effects analysis for ANS growth with FI, VSWM and PS as moderators.

	Model 6 (FI moderates growth)	Model 7 (VSWM moderates growth)	Model 8 (PS moderates growth)
**Constant**	-.17* (.07)	-.13 (.07)	-.14* (.07)
**Time**	.15* (.07)	.10 (.07)	.13 (.07)
**Time**^**2**^	-.04* (.02)	-.03 (.02)	-.04 (.02)
**FI**	.05 (.04)	.12*** (.03)	.13*** (.03)
**VSWM**	.13*** (.03)	.10* (.04)	.12 (.03)
**PS**	-.10*** (.03)	-.10*** (.03)	-.03 (0.04)
**NL**	.12*** (.03)	.12*** (.03)	.12*** (.03)
**Country**	.17** (.07)	.15* (.07)	.14* (.07)
***Interaction effects***			
Time*FI	.06** (.02)		
Time*VSWM		.02 (.02)	
Time*PS			-.05* (.02)
**Intercept variance**	.35	.35	.35
**Residuals**	.49	.49	.49
**Slope variance (time)**	.05	.05	.05
**Covariance between intercept and slope (time)**	-.06	-.07	-.07
**Log likelihood**	-2138.42	-2141.19	-2138.68
**LR test (Δdf)**	6.18* (1) (vs. Model 5)	.64 (1) (vs. Model 5)	5.65* (1) (vs. Model 5)

***p < .001

** *p* < .01

*p < .05

**Fig 3:**
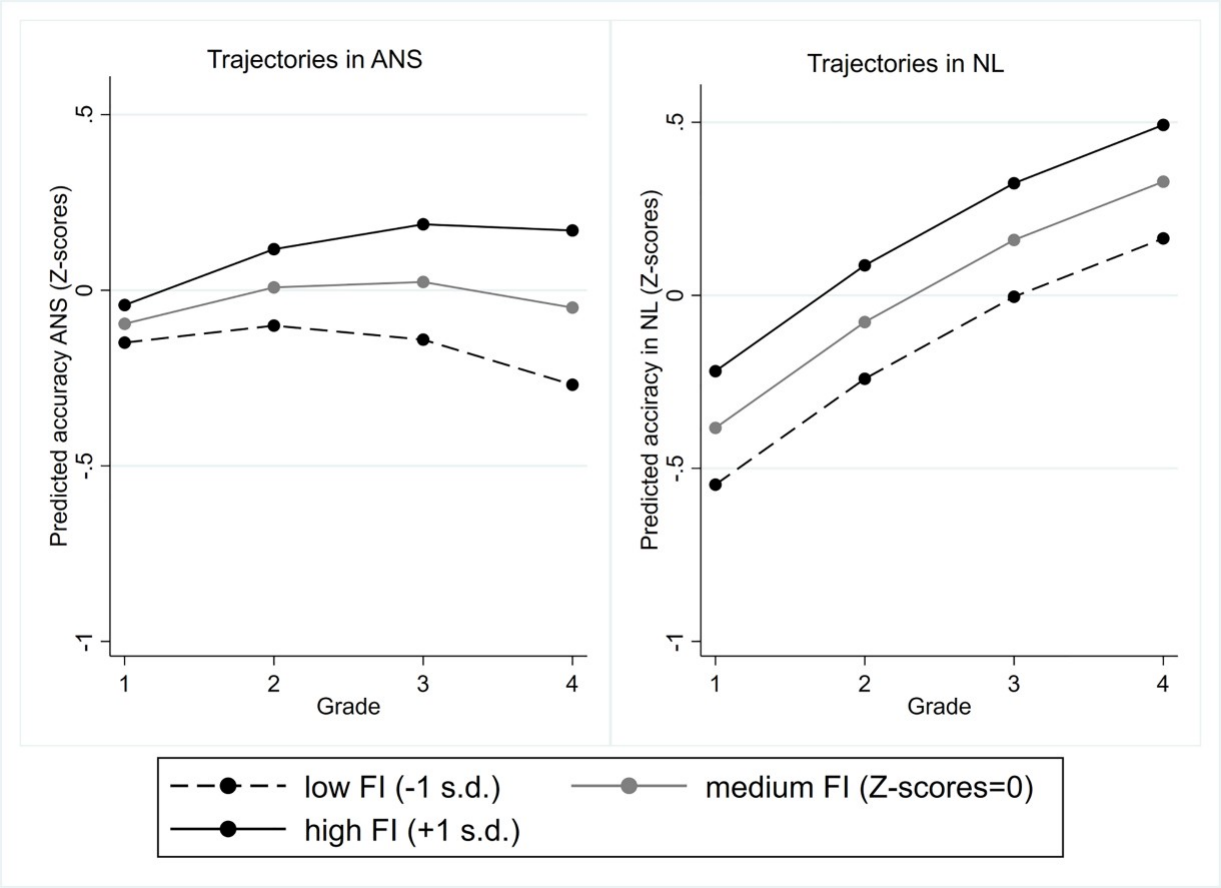
Average developmental trajectories in ANS and NL accuracy in pupils with different levels of FI.

The results of Model 6 for ANS revealed that time changes in ANS varied across pupils with different levels of FI (Fig 3). In particular, growth in accuracy was significant for pupils with high FI and not significant for pupils with low FI (Table 7).

**Table 7:** Results of simple slope analysis for growth in ANS for different levels of FI.

Level of FI	The coefficient of time variable	95% CI
**Low FI (-1 s.d.)**	0.09 (0.06)	-0.09; 0.16
**Medium FI (mean)**	0.15* (0.07)	0.02; 0.28
**High FI (+ 1 s.d.)**	0.20** (0.08)	0.05; 0.36

** *p* < .01

*p < .05

The coefficient for FI also changed, which indicated that the association between FI and ANS accuracy increased over time and that the difference in ANS accuracy depending on FI also increased.

In Model 7, the interaction term between VSWM and time variable was not significant. These data indicated that time changes in ANS accuracy did not vary depending on VSWM and that the effect of VSWM was stable across grades.

**Fig 4:**
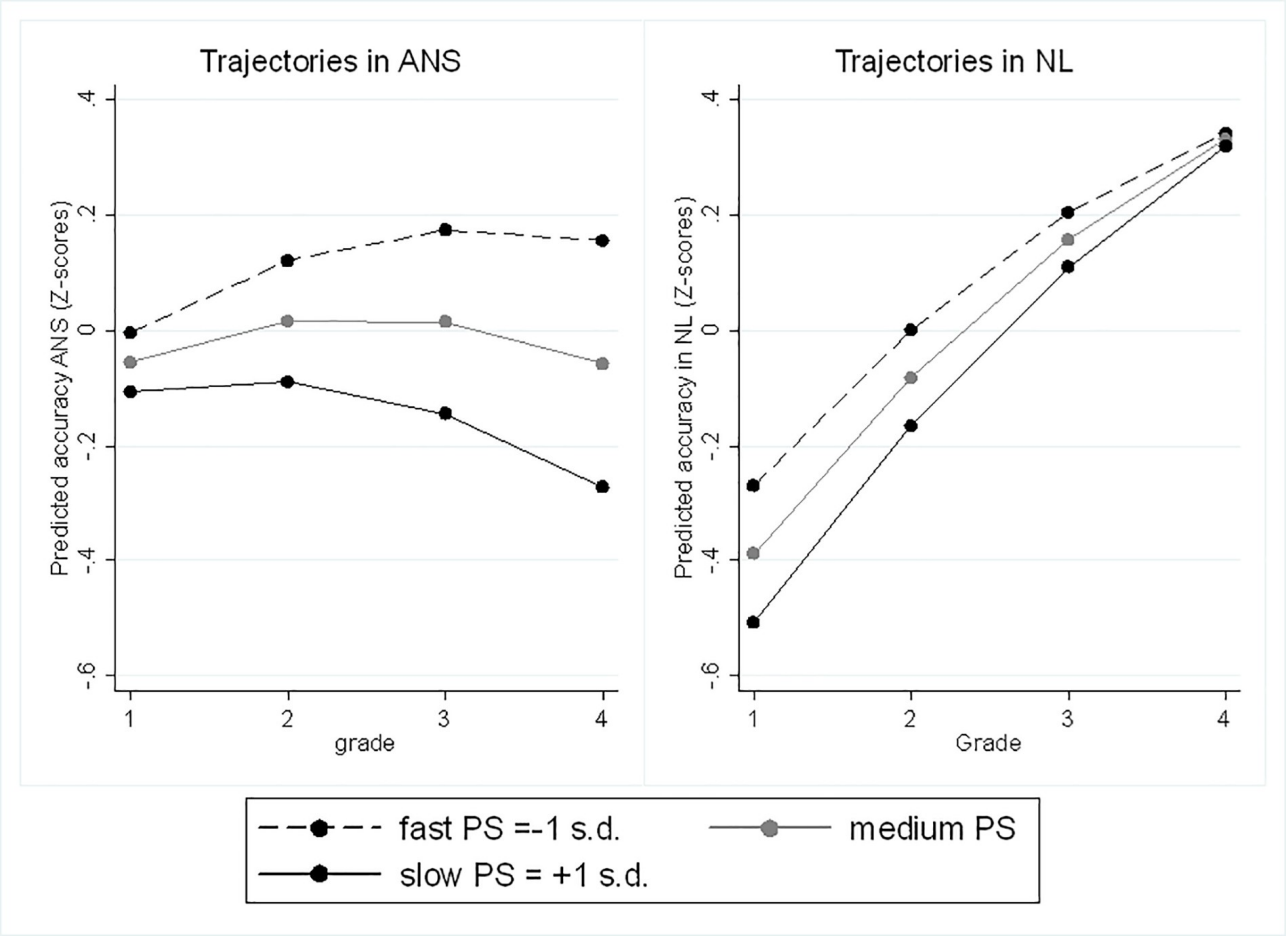
Average developmental trajectories in ANS and NL accuracy in pupils with different levels of PS.

In Model 8, the interaction between PS and time variable was significant and negative, indicating that time changes in ANS varied depending on the level of PS (Fig 4). The growth in ANS was significant for individuals with faster PS (or low reaction time) (Table 8). The results also demonstrated that the association between PS and NL increased across grades.

**Table 8:** Results of simple slope analysis for growth in ANS for different levels of PS.

Level of PS	The coefficient of time variable	95% CI
**Fast PS (-1 s.d.)**	0.18* (0.07)	0.03; 0.32
**Medium PS (mean)**	0.13 (0.07)	-0.002; 0.25
**Slow PS (+ 1 s.d.)**	0.07 (0.06)	-0.05; 0.20

*p < .05

Results of the analysis for NL as outcome are presented in Table 9.

**Table 9:** Results of mixed-effects analysis for NL with FI, VSWM and PS as moderators.

	Model 6 (FI moderates growth)	Model 7 (VSWM moderates growth)	Model 8 (PS moderates growth)
**Constant**	-.42*** (.07)	-.39*** (.07)	-.40*** (.07)
**Time**	.38*** (.07)	.34*** (.06)	.34*** (.06)
**Time**^**2**^	-.04* (.02)	-.03 (.02)	-.03 (.02)
**FI**	.17*** (.04)	.16*** (.03)	.16*** (.03)
**VSWM**	.09*** (.02)	.16*** (.04)	.10*** (.02)
**PS**	-.06* (.03)	-.06* (.03)	-.12** (.04)
**ANS**	.11*** (.02)	.11*** (.02)	.11*** (.02)
**Country**	.004 (.05)	.006 (.05)	.02 (.05)
***Interaction effects***			
Time*FI	-.004 (.02)		
Time*VSWM		-.03 (.02)	
Time*PS			.04 (.02)
**Intercept variance**	.70	.69	.70
**Residuals**	.42	.42	.42
**Slope variance (time)**	.05	.05	.05
**Covariance between intercept and slope (time)**	-.20	-.19	-.19
**Log likelihood**	-1961.86	-1960.25	-1960.07
**LR test (Δdf)**	.04 (1) (vs. Model 5)	3.28 (1) (vs. Model 5)	3.62 (1) (vs. Model 5)

***p < .001

** *p* < .01

*p < .05

The results of the analysis of interactions between the time variable and cognitive predictors for NL accuracy revealed that the growth did not significantly vary across students with different levels of FI (Fig 3), VSWM and PS (Fig 4). These data also indicated that the association between cognitive predictors and NL accuracy did not change across the four grades.

## Discussion

The first aim of this study was to estimate and compare growth trajectories in nonsymbolic and symbolic magnitude representations across four years of elementary school. Nonsymbolic representation was explored with the nonsymbolic comparison test (“blue-yellow dot test”), and symbolic representation was explored with the NL test. Previous studies have demonstrated that experience in formal education was associated with the growth in accuracy of both nonsymbolic and symbolic magnitude representations and that accuracy in symbolic representation improved to a greater extent than that in nonsymbolic representation [58].

In accordance with previous studies, our analysis demonstrated that growth in ANS accuracy was lower than that in NL accuracy across the four years of elementary school. The analysis also revealed differences between symbolic and nonsymbolic representations in intra-individual variability. The ICC for ANS accuracy was 0.37, and that for NL accuracy was 0.24. The ICC in the mixed-growth models reflected the proportion of inter-individual variance to common variance in outcome. The higher value of ICC might indicate that any outcome can be considered as a time-stable trait, which varied to a greater extent between individuals but not within individuals at different time points. From this point of view, the ANS is a more stable trait and showed more variability between individuals than NL performance. These results are in line with the studyof [84], who, demonstrated that performance on the ANS test was relatively stable in pre-school year [84]. Although their findings related to pre-school children, it is possible that stability in the ANS may extend into elementary school years.

Examination of individual differences in nonsymbolic representation revealed that the intra-individual differences in accuracy of nonsymbolic representations did not change over time. Pupils might demonstrate significant growth in accuracy, but their rank within the sample remained the same. Unlike nonsymbolic representation, the variability in symbolic representation reduced across the years in elementary school. Therefore, pupils who had a lower accuracy in symbolic representation at the start of formal education might demonstrate higher growth than students with high accuracy. Notably, the variance of intercept that referred to variability in accuracy at grade 1 was larger for symbolic representation than for nonsymbolic representation. Later in development, the variability in accuracy with symbolic representations decreased and became smaller than the variability in accuracy with nonsymbolic representations.

It is possible that the differences in the rate of changes of symbolic and nonsymbolic representation might be partly explained by differences in instructional aims and practices regarding the usage of symbolic and nonsymbolic skills in elementary school in Russia and Kyrgyzstan. The notable growth in the precision of symbolic representation might be associated with the intensive acquisition of symbolic number knowledge in elementary school. The instruction in elementary school in Russia and Kyrgyzstan has focused on the development of symbolic number knowledge, and teachers often use the concept of number lines to teach pupils. At the same time, the development of nonsymbolic magnitude representation has not been the aim of instruction in elementary school both in Russia and Kyrgyzstan. Teachers rarely used nonsymbolic skills and tasks that require the direct application of nonsymbolic skills.

The second aim of the current study was to estimate the extent to which growth in ANS and NL accuracy might be explained by growth in domain-general cognitive functions such as FI, VSWM and PS. Previous studies have demonstrated that FI and certain components of WM correlated with both nonsymbolic and symbolic magnitude representation and math achievement [85]. At the same time, little is known about how these cognitive predictors relate to the growth in accuracy in symbolic and nonsymbolic representations.

Our results revealed that the growth in the accuracy of nonsymbolic magnitude representation was explained by growth in FI, VSWM and PS, whereas growth in NL accuracy remained significant when developmental changes in cognitive predictors were taken into account. Considering the growth in cognitive predictors, nonsymbolic representation was stable across time. This finding confirmed our previous conclusions regarding the greater malleability of symbolic representations compared with nonsymbolic representations.

Some previous studies have demonstrated that ANS accuracy significantly increased (e.g., [21, 22, 86]). In particular, it was shown that the precision of the ANS measured by the Weber fraction continued to increase through childhood and adulthood [22]. Moreover, another study demonstrated that peak levels of precision were attained late in adulthood, near thirty years of age [21].

It is possible to suggest several possible reasons for the differences between our findings and other studies. First, most studies on the development of ANS accuracy used cross-sectional designs [21, 85]. Unlike longitudinal studies, using cross-sectional data did not allow an accurate estimation of the growth trajectories. Second, these studies did not take into account the growth in other cognitive functions, such as FI or WM. It might be the case that, for example, the difference between children and adults in the acuity of ANS was explained by differences in intelligence or working memory. The seeming growth in accuracy of the ANS might be an indicator of the growth in intelligence, working memory or PS. According to our results, at least in elementary school, this is true.

Another possible reason for the difference between our findings and previous results is characteristics of the samples. Our sample consisted of pupils from Russia and Kyrgyzstan. An estimation of cross-country differences in growth of ANS revealed that the rate growth was significantly lower in the Kyrgyz sample [24]. These differences might be partly explained by certain characteristics of educational processes in elementary school in the Kyrgyz sample Particularly, the class size was larger in the school in Kyrgyzstan than in Russia. In the Russian sample, the class size varied from 19 to 27, whereas in Kyrgyzstan, the class size varied from 40 to 45 students. Previous studies revealed that in large classes, pupils receive less help from the teacher than in small classes, and teachers tend to use more directive instructions and formal assessment [87–89]. In addition, teachers in the Kyrgyz school had to work in two shifts and had to prepare for lessons in two different classes with different curricula, and they had to grade much more homework. A large class size and overworked teachers can lead to ineffective feedback in the process of education. The lack of effective feedback may have prevented the improvement in precision of ANS in the Kyrgyz sample. Therefore, the lower growth in nonsymbolic representation in our study might be partly explained by the large proportion of pupils who experienced no growth in ANS. It should be noted that our study is not population-based and that results were obtained from two schools only. Thus, results regarding cross-country differences should be generalized to the entire population with caution.

We also tested the hypothesis that the rate of changes in ANS and NL accuracy varied across pupils with different levels of FI, VSWM or PS. Our analysis revealed that FI and PS moderate the rate of growth in ANS accuracy. Although the growth in ANS was nonsignificant when controlling for FI, VSWM and PS, the analysis of models with interaction revealed that the growth was significant for pupils with a high level of FI or with fast PS.

Some studies demonstrated the close relationship between FI and PS [70, 90, 91]. According to these findings, our results revealed that patterns of association between PS and FI with ANS were very similar. The association between the rate of growth of ANS precision and the level of FI or PS might reflect the existence of overlap between ANS and general cognitive ability. Genetic behavioral studies revealed that there exists generalist genetic overlap among general cognitive skills and ANS [72]. In contrast with ANS, growth in NL accuracy did not vary across pupils with different levels of general cognitive predictors.

FI might also be associated with the rate of growth in ANS due to shared perceptual skills. There is evidence that Raven’s SPM test measures not only general intellect (g factor) but also other abilities, such as perceptual or spatial abilities [92–94]. Relations between the development of nonsymbolic representations and FI might indicate that the growth in precision of nonsymbolic representations might occur as a consequence of a more precise estimation of the visual properties of the sets of objects that need to be compared. It had been previously shown that children can rely on a comparison of the total surface area or convex hull between two compared sets of objects to make comparison judgments [95–97]. All stimuli used in our study were congruent (the larger set had the larger surface area), so individuals with higher FI or perceptual ability could evaluate and compare surface area more precisely and, consequently, demonstrated significant growth in accuracy with the nonsymbolic comparisons. Using the version of the ANS test with congruent trials only is one limitation of the current study. Future studies are needed to test the hypothesis that developmental changes in the accuracy of nonsymbolic comparisons in incongruent trials might be less dependent on the growth in FI.

In summary, our study demonstrated that growth in symbolic accuracy was a more universal phenomenon in elementary school, while growth in nonsymbolic representation was explained by growth in FI, PS and VSWM and was dependent on the level of FI or PS. Results demonstrated that although changes in symbolic and nonsymbolic representations correlated across development, the two systems of magnitude representations are distinct since they demonstrated different patterns of development and different associations with domain-general cognitive functions. Future studies are needed to clarify developmental differences between nonsymbolic and symbolic representations and their relations with other cognitive functions in the secondary and high school years. The ongoing longitudinal project CLASS gives us this opportunity.

## Supporting information

S1 Table Mean accuracy and 95% confidence interval in ANS test for different ratio bins. (DOC)

S2 Table Correlations between measures for each grade. (DOCX)
