# Supplementary material for: Domain-general cognitive functions fully explained growth in nonsymbolic magnitude representation but not in symbolic representation in elementary school children
Source: PLoS One. 2020 Feb 11;15(2):e0228960. doi: 10.1371/journal.pone.0228960 (PMC7012440; doi:10.1371/journal.pone.0228960)
Supplement: S1 Table — (DOC) [file pone.0228960.s001.doc]

**Supporting information**

**S1 Table 1. Mean accuracy and 95% confidence interval in ANS test** for different ratio bins

| **Ratio** | **Grade 1** | **Grade 2** | **Grade 3** | **Grade 4** |
| --- | --- | --- | --- | --- |
| 0.30 – 0.66 | .71 [.70; .72] | .74 [.73; .75] | .77 [.76; .78] | .79 [.78; .80] |
| 0.67 – 0.72 | .64 [.63; .65] | .66 [.65; .67] | .67 [.66; .68] | .69 [.68; .70] |
| 0.73 – 0.78 | .59 [.58; .60] | .61 [.60; .62] | .64 [.63; .65] | .65 [.64; .66] |
| 0.79 – 0.84 | .56 [.56; .57] | .58 [.57; .59] | .60 [.59; .61] | .60 [.59; .61] |
| 0.85 – 0.87 | .57 [.56; .58] | .59 [.58; .60] | .60 [.59; .61] | .61 [.60; .62] |

**S2 Table 2**. Correlations between measures for each grade

|  | **ANS** | **NL** | **VSWM** | **PS** |
| --- | --- | --- | --- | --- |
| **Grade 1** | | | | |
| **ANS** | 1 |  |  |  |
| **NL** | .16* | 1 |  |  |
| **VSWM** | .17* | .26*** | 1 |  |
| **PS** | -.20* | -.19* | -.29*** | 1 |
| **FI** | .26*** | .35*** | .37*** | -.29*** |
| **Grade 2** | | | | |
| **ANS** | 1 |  |  |  |
| **NL** | .19** | 1 |  |  |
| **VSWM** | .24*** | .31*** | 1 |  |
| **PS** | -.14 | -.14* | -.27*** | 1 |
| **FI** | .35*** | .23** | .25*** | -.25*** |
| **Grade 3** | | | | |
| **ANS** | 1 |  |  |  |
| **NL** | .22** | 1 |  |  |
| **VSWM** | .29*** | .33*** | 1 |  |
| **PS** | -.17* | -.18** | -.27*** | 1 |
| **FI** | .32*** | .34*** | .39*** | -.09 |
| **Grade 4** | | | | |
| **ANS** | 1 |  |  |  |
| **NL** | .39*** | 1 |  |  |
| **VSWM** | .23*** | .31*** | 1 |  |
| **PS** | -.32*** | -.28*** | -.30*** | 1 |
| **FI** | .38*** | .35*** | .41*** | -.18* |

****p*<.001; ***p*<.01, **p*<.05
