# Supplementary material for: Domain-general cognitive functions fully explained growth in nonsymbolic magnitude representation but not in symbolic representation in elementary school children
Source: PLoS One. 2020 Feb 11;15(2):e0228960. doi: 10.1371/journal.pone.0228960 (PMC7012440; doi:10.1371/journal.pone.0228960)
Supplement: S2 Table — (DOCX) [file pone.0228960.s002.docx]

**S2 Table 2. Correlations between measures for each grade**

|  | **ANS** | **NL** | **VSWM** | **PS** |
| --- | --- | --- | --- | --- |
| **Grade 1** | | | | |
| **ANS** | 1 |  |  |  |
| **NL** | .16* | 1 |  |  |
| **VSWM** | .17* | .26*** | 1 |  |
| **PS** | -.20* | -.19* | -.29*** | 1 |
| **FI** | .26*** | .35*** | .37*** | -.29*** |
| **Grade 2** | | | | |
| **ANS** | 1 |  |  |  |
| **NL** | .19** | 1 |  |  |
| **VSWM** | .24*** | .31*** | 1 |  |
| **PS** | -.14 | -.14* | -.27*** | 1 |
| **FI** | .35*** | .23** | .25*** | -.25*** |
| **Grade 3** | | | | |
| **ANS** | 1 |  |  |  |
| **NL** | .22** | 1 |  |  |
| **VSWM** | .29*** | .33*** | 1 |  |
| **PS** | -.17* | -.18** | -.27*** | 1 |
| **FI** | .32*** | .34*** | .39*** | -.09 |
| **Grade 4** | | | | |
| **ANS** | 1 |  |  |  |
| **NL** | .39*** | 1 |  |  |
| **VSWM** | .23*** | .31*** | 1 |  |
| **PS** | -.32*** | -.28*** | -.30*** | 1 |
| **FI** | .38*** | .35*** | .41*** | -.18* |

****p*<.001; ***p*<.01, **p*<.05
